# Effects of Electromagnetic Radiation on Neuropeptide Transcript Levels in the Synganglion of *Ixodes ricinus*

**DOI:** 10.3390/pathogens12121398

**Published:** 2023-11-28

**Authors:** Lívia Šofranková, Miroslav Baňas, Natália Pipová, Igor Majláth, Juraj Kurimský, Roman Cimbala, Marek Pavlík, Lourdes Mateos-Hernández, Ladislav Šimo, Viktória Majláthová

**Affiliations:** 1 Department of Animal Physiology, Pavol Jozef Šafárik University in Košice, Šrobárova 2, 04180 Košice, Slovakia; livia.sofrankova@student.upjs.sk (L.Š.); miroslav.banas@student.upjs.sk (M.B.); natalia.kokosova@upjs.sk (N.P.); igor.majlath@upjs.sk (I.M.); 2 Department of Electrical Power Engineering, Faculty of Electrical Engeneering and Informatics, Technical University of Košice, Mäsiarska 74, 04120 Košice, Slovakia; juraj.kurimsky@tuke.sk (J.K.); roman.cimbala@tuke.sk (R.C.); marek.pavlik@tuke.sk (M.P.); 3 Laboratoire de Santé Animale, Unitè Mixte de Recherche de Biologie Molèculaire et d’Immunologie Parasitaires (UMR BIPAR), Ecole Nationale Vétérinaire d’Alfort, INRAE, ANSES, F-94700 Maisons-Alfort, France; lourdes.mateos@vet-alfort.fr (L.M.-H.); ladislav.simo@vet-alfort.fr (L.Š.)

**Keywords:** ticks, neuropeptides, transcript levels, *Ixodes ricinus*

## Abstract

Anthropogenic electromagnetic radiation is an important environmental factor affecting the functionality of biological systems. Sensitivity to various frequencies of electromagnetic radiation has been detected in ixodid ticks in the past. However, the physiological aspects of radiation effects have not yet been studied in ticks. In the presented experiment, 360 *Ixodes ricinus* ticks, 180 males and 180 females, were divided into 16 irradiated and 8 control groups. The irradiated groups were exposed to two different intensities of electromagnetic radiation with a frequency of 900 MHz at different lengths of exposure time. RT-PCR was utilized to determine the changes in mRNA levels in tick synganglia after irradiation. Four randomly selected neuropeptide genes were tested—allatotropin (*at*), FGLa-related allatostatins (*fgla*/*ast*), kinin, and arginine-vasopressin-like peptide (*avpl*). A significant decrease in transcript levels in all female groups exposed to higher intensity radiofrequency radiation for 1 to 3 h was found. After one hour of radiofrequency exposure, a significant downregulation in allatotropin expression in males was detected. A consistent downregulation of the *at* gene was detected in males irradiated with at a higher intensity. Unfortunately, the specific functions of the studied neuropeptides in ticks are not known yet, so a more comprehensive study is necessary to describe the effects of EMF on observed neuropeptides. This study represents the first report on the effects of the abiotic environment on tick neurophysiology.

## 1. Introduction

Natural and anthropogenic electromagnetic fields (EMFs) are an important environmental factor affecting all living organisms. Among those, anthropogenic electromagnetic field, commonly labeled as electromagnetic smog, is a significant environmental pollutant affecting a wide range of cell and live system functions. Studies of the effects of EMFs and electromagnetic perception mechanisms have been conducted on in vitro cultures and in several invertebrate and vertebrate laboratory models [1,2,3,4,5,6,7,8]. It has been well documented that electromagnetic radiation affects the structure of the lipid bilayer, the polarity of membranes, and the sensitivity of ion channels [9,10,11,12,13,14]. The presence of EMFs can also cause the disturbance of the cell’s electrochemical equilibrium, which subsequently induces the activation of the stress response [15]. Furthermore, electromagnetic fields influence the cell at the molecular level, which involves, for example, the gene expression and subsequent proteosynthesis. Among those, the upregulation of heat shock proteins (HSP) and proteins involved in the immune response, cell proliferation, and cell apoptosis has been demonstrated [16,17,18,19,20,21,22]. In *Drosophila melanogaster*, radiation influenced the levels of biogenic amines in the central nervous system [23].

Pioneer studies describing the influence of electromagnetic radiation on ticks were published by Korotkov et al. (1996, 2000). Here the authors proved that microwave radiation exposure affected the hatching time, activity, and survival of *Hyalomma asiaticum* tick larvae. The experimental temperature during radiation exposure appeared to be an important factor of EMF influence in this study [24,25]. In addition, one-hour radiofrequency irradiation of *Dermacentor reticulatus* eggs resulted in the hatching of larvae with larger body dimensions in later studies [26]. The most recent studies investigating EMF-mediated changes in tick behavior have clearly demonstrated the ability of ticks to perceive electromagnetic fields and distinguish their different properties [27,28]. Interestingly, the presence of bacteria (*Rickettsia* spp.) in ticks was associated with enhancing the response of infected ticks to radiation [29]. The physiological basis of the behavioral responses observed in irradiated ticks has not been explained. However, as it is most likely mediated by the central nervous system, the levels of neurotransmitters and neuropeptides could have a role in the regulation of the behavior observed under irradiation. 

Neuropeptides are small protein molecules acting as crucial regulators of arthropod physiology and behavior [30]. They are well known to be expressed in specific neuronal or neuroendocrine cells regulating a myriad of physiological processes, e.g., feeding, development, reproduction, homeostasis, growth, digestion, diuresis, sleeping, or stress. In addition, these signaling molecules are well known for acting as neuromodulators in the circuits of the central nervous system [31]. As of today, in the tick central nervous system, the synganglion, 38 different neuropeptide genes have been identified [32,33]. Among those, multiple have been immunolocalized in distinct neuronal cells within the synganglion as well as in their axons reaching several visceral organs [34,35,36,37]. Although the knowledge regarding the characterization and localization of tick neuropeptides along their receptors has accelerated in the last 15 years, most of their functions remain obscured. 

In the presented study, we investigated the influence of different 900 MHz EMF intensities on selected neuropeptide transcripts—allatotropin (*at*), FGLa-related allatostatins (*fgla*/*ast*), kinin, and arginine-vasopressin-like peptide (*avpl*) in adult-stage male and female *I. ricinus* synganglia. 

## 2. Materials and Methods

### 2.1. Experimental Ticks

**Figure 1:**
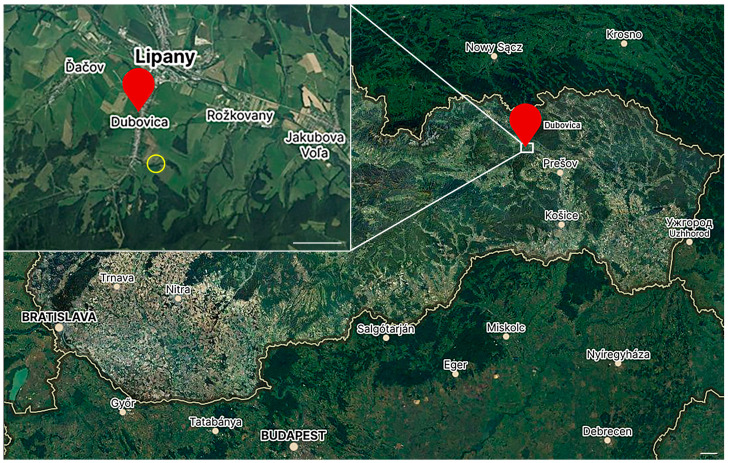
A map of tick collection site. Ticks were collected in northeastern Slovakia (map scale = 10 km), near the village Dubovica (GPS: 49°07′3.7″ N 20°57′37.2″ E). The inset figure shows the village (insert figure scale = 1 km); the yellow circle marks the collection site. Source of maps: “https://en.mapy.cz/” (accessed on 26 October 2023).

Altogether, 360 adult-stage sheep ticks (*Ixodes ricinus*), 180 males and 180 females, were used. Ticks were collected via flagging near the village Dubovica, northeastern Slovakia (GPS coordinates 49°07′3.7″ N 20°57′37.2″ E). Ticks were collected in the spring (April–May) of 2022 on a meadow, its slope oriented towards the northeast. Ticks were collected in the shrubbery with many signs of animal tracks, located on the edge of a brook running along the perimeter of the meadow (Figure 1). The data on the intensity of electromagnetic fields were not available for Dubovica; there was a radio tower of a mobile phone provider (O_2_ Slovakia) in Lipany, approximately 1.5 km from the collection site [38]. The intensity of the EMF in the nearest town, Sabinov (13 km distance), is, according to available data, 0.25–0.3 V/m [39]. Collected ticks were kept under a natural light regime in the laboratory desiccator to ensure proper constant humidity conditions. 

### 2.2. Irradiation Procedure

Ticks were divided into 24 experimental groups, 5 ticks per group. To ensure the proper hydration of ticks, each group was placed in a plastic 2 mL tube with moistened filter paper before irradiation. The irradiation was conducted in an anechoic chamber (model 1710–100, Comtest Engineering, Leyde, The Netherlands), ensuring no external electromagnetic fields affected the experiment. A signal generator (N5183A, Agilent Technologies, Kuala Lumpur, Malaysia) was used as a source of radiofrequency electromagnetic field (RF EMF) connected to a Double-Ridged Waveguide Horn Antenna HF907 (Rohde and Schwarz, Munich, Germany). The antenna was placed approximately 2 m from the experimental apparatus containing ticks.

During the experiment, ticks were irradiated (Table 1) with a constant, polarized, unmodified electromagnetic field at a 900 MHz frequency and two different intensities—2 V/m and 40 V/m. The duration of the exposure was 10 min, 1 h, 3 h and 24 h. Together, 4 control groups of ticks were placed into the anechoic chamber for the designated times but without irradiation. 

### 2.3. Dissection Protocol

Immediately after irradiation, ticks were dissected live under a stereomicroscope. Ticks were individually placed on double-sided tape, ventral side down. Ticks were cut along the alloscutum using the tip of a scalpel, and the dorsal part of the cuticle was removed. A drop of ice-cold phosphate-buffer saline (PBS; 137 mM NaCl, 1.45 mM NaH_2_PO_4_, 20.5 mM Na_2_HPO_4_, pH 7.2) was added onto the exposed organs. Synganglia were surgically removed using precision tweezers with super-fine tips (type #5SF, DUMONT SWITZERLAND, Courtemaîche, Switzerland) and placed into a separate drop of PBS. Synganglia were cleaned from accessory tissues and washed in several drops of clean PBS. Tissues were flash-frozen in 1.5 mL tubes on dry ice. 

### 2.4. RNA Isolation of Tick Synganglia and Quantitative Real-Time Reverse Transcriptase PCR (qRT-PCR)

A pool of 5 synganglia was used for each experimental condition. Total RNA was isolated using an RNeasy^®^ Micro Kit (Qiagen, Venlo, The Netherlands). The concentration and purity of obtained RNA were measured using a NanoDrop ONE (Thermo Scientific, Waltham, MA, USA). The obtained RNA was reverse-transcribed to cDNA using a RevertAid H Minus First Strand cDNA Synthesis kit (Thermo Scientific), using oligo (dT) primers. To determine the change in the mRNA levels, qRT-PCR was performed using a LightCycler^®^ 480 II (Roche, Meylan, France) thermocycler. LightCycler^®^ 480 SYBR^®^ Green I Master mastermix (Roche) was used each 20 μL reaction was prepared from 10 μL of mastermix, 7 μL of PCR-grade H_2_O, 1 μL of 10 μM forward and 1 μL of 10 μM reverse primer, and 1 μL of template cDNA. Primers for qRT-PCR are listed in Table 2. The nomenclature of studied neuropeptides and their genes is according to Coast and Schooley 2011 [40]. As a reference gene, the ribosomal protein S4 (*rps4)* was used [41].

Opaque 96-well plates were used for RT-PCR. The preincubation step of the cycling protocol was carried out at 95 °C for 5 min, followed by the amplification step at 95 °C (10 s), 60 °C (for *fgla*/*ast* and *at*) or 55 °C (for *kinin* and *avpl*) (10 s), and 72 °C (10 s). A total of 45 cycles were used for the amplification step. The melting curve was analyzed from 65 °C to 97 °C. The amplicons of targeted genes were verified via sequencing the qRT-PCR products (Eurofins, Luxembourg, Luxembourg). 

Relative transcript levels were calculated utilizing the ΔΔCt ratio method according to Livak and Schmittgen, 2001 using Microsoft Excel and are expressed as fold change value [43]. Graphs for each studied gene were created in GraphPad 5 Prism (GraphPad Software Inc., San Diego, CA, USA). Statistical significance was assessed using Student’s *t*-test in GraphPad 5 Prism (GraphPad Software Inc.). This experiment was conducted in 3 biological replications and in two technical replications.

## 3. Results

**Figure 2:**
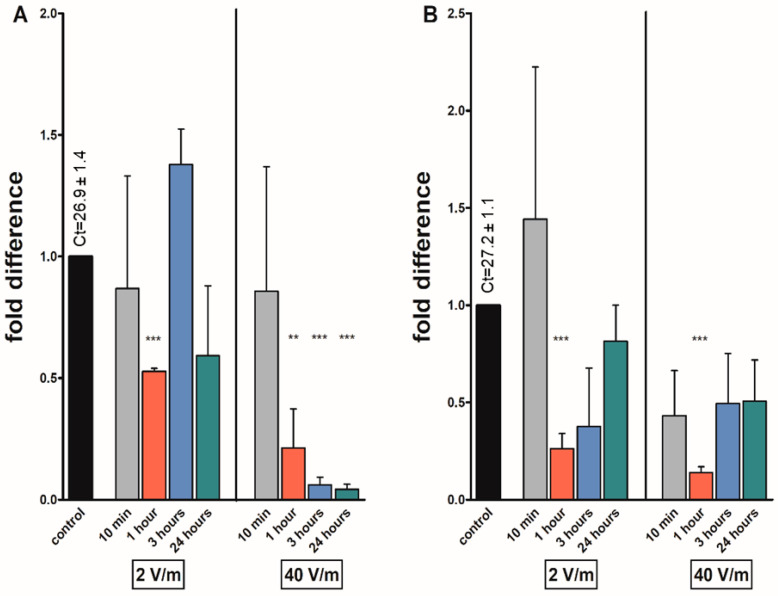
Effects on different EMF intensities on the allatotropin (*at*) transcripts in *I. ricinus* adult synganglia of (**A**) females; (**B**) males. Data were normalized using the ribosomal protein S4 transcript (*rps4*), and the expression levels in non-irradiated ticks were assigned a value of 1. Results shown are means and standard errors of means. Asterisks indicate the comparison of the mean to the value of the non-irradiated group. The statistical significance of the fold difference was calculated using an unpaired *t*-test and is expressed using *p* values: ** *p* < 0.01; *** *p* < 0.001.

Reduced mRNA levels of *at* were found in most of the irradiated groups, significantly after one hour of irradiation in both sexes. In females, the levels were reduced 0.53-fold and 0.21-fold after one-hour exposure. In male groups, the levels were reduced 0.26 and 0.14 folds after 1 h exposure under same experimental conditions. In females exposed to 40 V/m radiation, the transcript levels of *at* were significantly reduced not only after the already-mentioned one-hour irradiation but also after 3 (0.06 folds) and 24 h (0.04 folds). The only increase in *at* levels, although statistically non-significant, was noticed after 10 min of 2 V/m exposure in males and 3 h in females (1.44 and 1.38 folds, respectively) (Figure 2).

**Figure 3:**
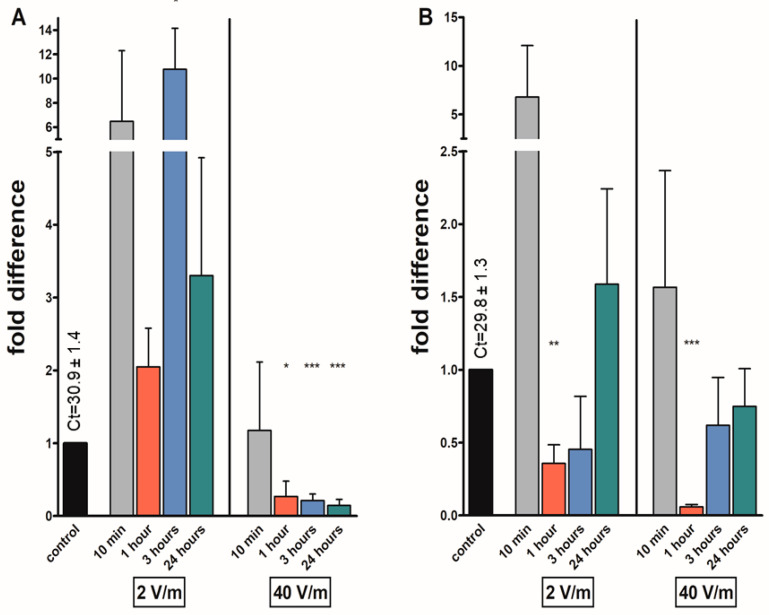
Effects on different EMF intensities on the FGLa-related allatostatin (*fgla*/*ast*) transcripts in *I. ricinus* adult synganglia from (**A**) females; (**B**) males. Data were normalized using the ribosomal protein S4 transcript (*rps4*), and the expression levels in non-irradiated ticks were assigned a value of 1. Results shown are means and standard errors of means. Asterisks indicate the comparison of the mean to the value of the non-irradiated group. The statistical significance of the fold difference was calculated using an unpaired *t*-test and is expressed using *p* values: * *p* < 0.05; ** *p* < 0.01; *** *p* < 0.001.

An elevation in mRNA levels of *fgla*/*ast* was found in all females irradiated with 2 V/m radiation, with fold changes ranging from 2.05 to 10.77. The 10.77-fold increase in the levels in females exposed to 2 V/m for 3 h was significant. In female groups exposed to 40 V/m for 1 h or more, *fgla*/*ast* levels were significantly reduced: 0.27 folds after 1 h, 0.21 after 3 h, and 0.14 folds after 24 h exposure. The levels of *fgla*/*ast* transcripts were reduced after 1 h (0.36-fold) and 3 h (0.45-fold) 2 V/m irradiation in males. In males exposed to a higher intensity, a decrease in mRNA levels after 1, 3, and 24 h of exposure was noted, but significantly only in the 1 h group (decrease of 0.05 folds) (Figure 3). 

**Figure 4:**
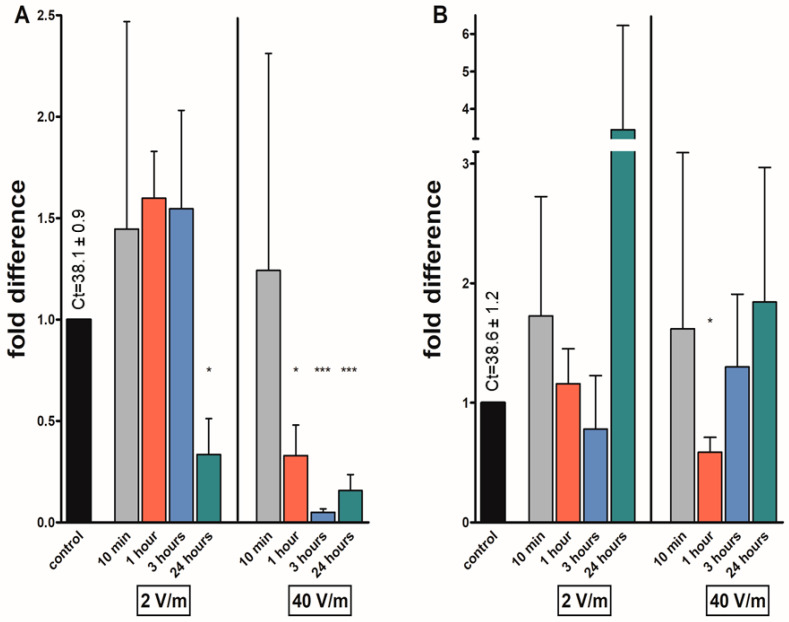
Effects on different EMF intensities on the arginine-vasopressin-like peptide (*avpl*) transcripts in *I. ricinus* adult synganglia from (**A**) females; (**B**) males. Data were normalized using the ribosomal protein S4 transcript (*rps4*), and the expression levels in non-irradiated ticks were assigned a value of 1. Results shown are means and standard errors of means. Asterisks indicate the comparison of the mean to the value of the non-irradiated group. The statistical significance of the fold difference was calculated using an unpaired *t*-test and is expressed using *p* values: * *p* < 0.05; *** *p* < 0.001.

The mRNA levels of *avpl* were significantly reduced 0.34-fold in the groups of females exposed to 2 V/m radiation for 24 h. The levels of *avpl* in the female groups exposed to 40 V/m reduced after 1 h 0.33 folds; after 3 h, 0.05 folds; and after 24 h of irradiation, 0.16 folds. In the rest of the female groups, the gene expression was upregulated, but the results were non-significant. A significant downregulation of transcripts was found in the group of males irradiated for one hour by 40 V/m (0.59-fold). The number of transcripts was slightly elevated in most male groups, however not significantly (Figure 4).

**Figure 5:**
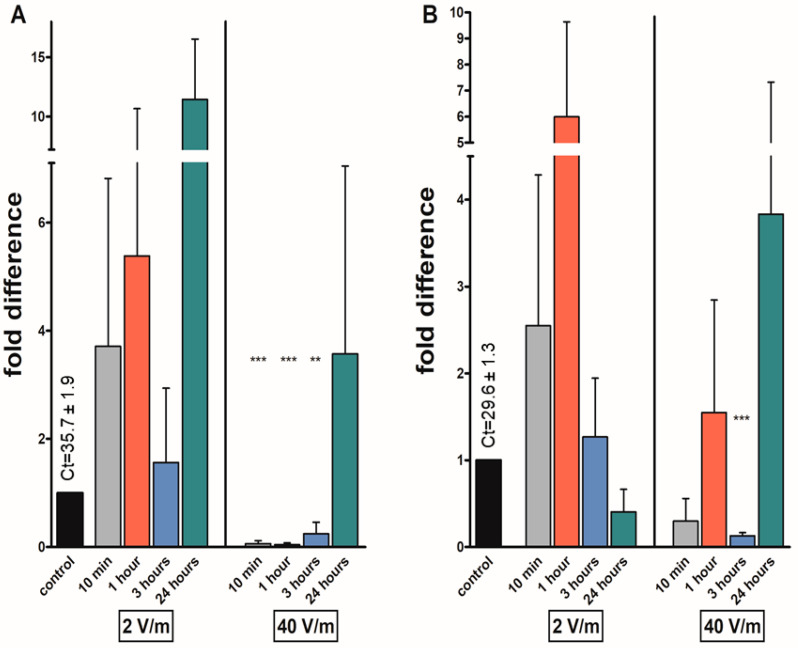
Effects on different EMF intensities on the *kinin* transcripts in *I. ricinus* adult synganglia from (**A**) females; (**B**) males. Data were normalized using the ribosomal protein S4 transcript (*rps4*), and the expression levels in non-irradiated ticks were assigned a value of 1. Results shown are means and standard errors of means. Asterisks indicate the comparison of the mean to the value of the non-irradiated group. The statistical significance of the fold difference was calculated using an unpaired *t*-test and is expressed as *p* values: ** *p* < 0.01; *** *p* < 0.001.

The mRNA levels of *kinin* were non-significantly increased in all groups of females irradiated with 2 V/m intensity (fold change from 1.56 to 11.44) and in the group exposed to 40 V/m radiation for 24 h (3.6-fold). Transcript levels were significantly reduced in the groups of females irradiated with the higher intensity for up to 3 h, specifically 0.06 folds after 10 min, 0.04 after 1 h, and 0.24 folds after 3 h of exposure. The levels of mRNA were slightly elevated in males during the first three hours of exposure to 2 V/m; however, these were non-significant results. When applying 40 V/m for 3 h, the levels of the *kinin* transcript were significantly reduced to 0.13-fold (Figure 5).

## 4. Discussion

In our experiment, a 900 MHz frequency—the mainstream spectrum for mobile data and voice broadband—was used to study the effect of electromagnetic radiation on selected transcript levels in the synganglion of both sexes of *I. ricinus*. This frequency has proven bioactive effects [7,44] and has been utilized in other tick behavioral tests and studies in the past. Several species of ticks (*I*. *ricinus*, *D*. *reticulatus*) showed a significant response to it [27,28,29]. In the questing arena set-up, *D. reticulatus* ticks displayed jerking movements [27]. In the modified T-labyrinth arena (RST test), ticks preferred the arm exposed to irradiation when a 900 MHz frequency was used for 24 h [27,28]. Female *I. ricinus* ticks tested in the same manner did not display any significant affinity for either arm. However, tracking ticks’ movement in the circular arena with irradiated and shielded parts revealed the difference in the movement dynamics [45]. 

We noted differences in the levels of neuropeptide mRNAs in the ticks’ synganglion after exposure to electromagnetic radiation with different intensities. Previous studies on drosophila and honeybees have reported different effects of radiofrequency radiation on the organism depending on the radiation parameters used as well [44,46,47,48]. Modulation, pulsed fields, intensity, and specific absorption rate have been cited as factors that enhance the biological effects of exposure [49]. In the irradiation study on drosophila, all intensities (power densities) produced a change in gene expression and enzyme activity after 48 h exposure, and it seems that there is still bioactive effect if the irradiation is less than 0.0613 V/m [50]. The effect of radiation, according to studies, is most noticeable from 30 cm distance but can still have an effect from a 1 m distance, especially if the power density is higher than 1 µW/cm [49].

In this study, an insignificant increase in the levels of transcripts was found in the lower-intensity radiation (2 V/m) female groups (Table 3). The 2 V/m intensity was utilized in this experiment, as it was found to be a common EMF intensity occurring in urban areas of eastern Slovakia [51]. It seems that the mRNA transcript levels are affected only slightly by lower-intensity radiation; however, utilizing higher intensity (40 V/m) produced an enhanced effect on the synganglion of *I. ricinus*. In females irradiated with 40 V/m, three out of four neuropeptide mRNA levels (*avpl*, *fgla*/*ast* and *at*) were significantly reduced after one hour of irradiation. Here, 40 V/m intensity was chosen for this experiment because it is close to the maximal 900 MHz radiation intensity allowed in Slovakia (which is 42 V/m [52]), although it can be rarely found in non-laboratory setups. As we hypothesize that EMF does affect neuropeptide levels, this intensity was chosen to simulate a supernormal stimulus. A much broader range of intensities and frequencies of EMF needs to be tested to acquire a more complex understanding of the effects of EMFs on the tick central nervous system in the future. 

The results for both intensities varied in male ticks (Table 4). Although previous reports indicate sex-specific expression of certain transcripts among tick sexes [53,54], at this point, it is difficult to speculate which molecular or physiological mechanisms caused the differences observed in our study. Despite this fact, our results are consistent with the behavioral differences detected among male and female ticks exposed to irradiation in our previous experiments. Specifically, in *D. reticulatus*, females expressed a specific jerking movement with the whole body, while male ticks jerked mostly with their front legs, so the same radiation exposure parameters produced a stronger response in females. In the RST arena (radiation-shielded tube arena), however, there was no significant preference for exposed/shielded arm between the sexes in *I. ricinus* and *D. reticulatus* [27,28,29]. Taken together, more studies involving multiple tick species are necessary to figure out the sex-specific responsiveness of male and female ticks to EMFs.

The duration of radiation exposure was also considered in this study. Behavioral studies on *D. reticulatus* and *I. ricinus* in the past reported that ticks preferred the exposed arm of the labyrinth after 24 h of exposure [28,29]. In our experiment, irradiation seemed to have had a significant effect on tick mRNA levels after at least 1 h of exposure. Interestingly, changes in locomotor behavior and an influence on larvae development after approximately one hour of exposure to 900 MHz were reported in ticks before. In a behavioral study of *I. ricinus* in EMF, ticks were irradiated by GSM mobile signal for 24 h and, after one hour, started spending a longer time walking in the exposed part of the experimental arena [43]. One hour of exposure to the same frequency significantly enlarged the body dimensions of *D. reticulatus* larvae hatched from irradiated eggs. From the groups of eggs exposed to radiation for 30 and 90 min, the larvae were smaller than the control larvae when hatched [27]. One hour of exposure therefore seems to be the minimal effective length of EMF irradiation for this frequency. 

Information about the effects of EMF on the arthropod nervous system is scarce. In previously published papers on drosophila, it is suggested that EMFs can affect the signaling pathways in ecdysteroid production [50] and the levels of biogenic amines in the brain [23]. Changes in biogenic amine levels after low-frequency irradiation were found in crickets as well [55]. The neuropeptide genes studied in this paper were selected randomly, as no previous studies on any neuropeptide genes in arthropods under influence of EMFs have been published so far. Here, a plausible approach for future studies would be mass sequencing, to obtain a more in-depth understanding of the changes in the synganglion transcripts under radiofrequency irradiation. Therefore, as our pioneer work investigated only four different neuropeptide transcripts, at this point, it is hard to ascertain the exact influence of irradiation on tick physiology and behavior that result from the orchestration of multiple gene products. 

The increasing prevalence of ticks and tick-borne pathogens in urbanized areas like city parks and recreational woods near the cities is currently an urgent problem [56,57]. Green spaces in cities provide suitable environmental conditions for ticks, plenty of hosts, and possibly a constant dose of EMF exposure. However, it is hard to conclude if the omnipresent anthropogenic fields provide some kind of specific stimulation for ticks, as much more research needs to be conducted on this topic. 

Since the first irradiation experiment published by Vargová et al. 2017, it was hypothesized that ticks could be able to perceive the inner electromagnetic field of their hosts [27]. Recently, it has been discovered that ticks likely utilize static electric fields to transport a very short distance through the air to latch onto their hosts while questing on vegetation. However, it was shown that this is a passive process of electrostatic attraction [58]. The question of the presence of some type of electromagnetic sense in ticks is still unanswered. Sensillae-like structures for infrared radiation in the capsule area of the Haller’s organ were described in *D. variabilis* [59]. Electromagnetic perception based on the presence of magnetite particles in the tissues found in bees and ants could also be a candidate mechanism of electromagnetic sensing in ticks [60,61]. 

## 5. Conclusions

To conclude this study, radiofrequency electromagnetic radiation does alter the levels of neuropeptide transcripts in the central nervous system of *Ixodes ricinus*. While both tested intensities affected the mRNA levels, the intensity of 40 V/m and 1 h of radiation exposure produced the most significant results. A more dramatic impact on female ticks was noted. Most EMF effects on transcript amount were gene-specific. 

We present a completely novel research focus among studies on the effects of the abiotic environment on the physiology of ticks. As the neuropeptidergic system exerts various functions in the tick body, a more extensive study of gene expression under irradiation conditions is required in the future.

## Figures and Tables

**Table 1:** Experimental groups created for the study.

Radiation Intensity	Biological Replications	Sex	Exposure Time	Number of Individuals
40 V/m	3	♀	10 min	5
			1 h	5
			3 h	5
			24 h	5
		♂	10 min	5
			1 h	5
			3 h	5
			24 h	5
2 V/m	3	♀	10 min	5
			1 h	5
			3 h	5
			24 h	5
		♂	10 min	5
			1 h	5
			3 h	5
			24 h	5
0 V/m	3	♀	10 min	5
			1 h	5
			3 h	5
			24 h	5
		♂	10 min	5
			1 h	5
			3 h	5
			24 h	5

**Table 2:** qRT-PCR primers used in this study.

Protein	Gene	Forward Primer (5′-3′)	Reverse Primer (5′-3′)	References
FGLa-related allatostatins	*fgla*/*ast*	AGCGGAGGTACAACTTTGGC	CTCCTCTTCCAGCGCTCG	[42]
Allatotropin	*at*	GGCTTTGGCAAGAGAATGAG	GGCTATTTCCTCCGCTAACC	[42]
Arginine-vasopressin like peptide	*avpl*	TCTCACACATGCTGCTCCTG	GCATCGTTGAGGATGCACAT	This study
Kinin	*kinin*	ACCACGTTCCTGATGAGCAT	ATGTTCCAGCGAATGAAGCT	This study
Ribosomal protein S4	*rsp4*	GGTGAAGAAGATTGTCAAGCAGAG	TGAAGCCAGCAGGGTAGTTTG	[41]

**Table 3:** Graphic depiction of the neuropeptide transcript levels in female experimental groups, comparison of the mean to the value of the non-irradiated group. The statistical significance of the fold difference was calculated using an unpaired *t*-test and is expressed with *p* values: grey color—no change, *p* > 0.5; green color—increase in mRNA level, *p* < 0.5; red color—decrease in mRNA level, *p* < 0.5; * *p* < 0.05; ** *p* < 0.01; *** *p* < 0.001.

♀	2 V/m				40 V/m			
Gene	10 min	1 h	3 h	24 h	10 min	1 h	3 h	24 h
*kinin*					***	***	**	
*avpl*				*		*	***	***
*at*		***				**	***	***
*fgla*/*ast*			*			*	***	***

**Table 4:** Graphic depiction of the neuropeptide transcript levels in male experimental groups, comparison of the mean to the value of the non-irradiated group. The statistical significance of the fold difference was calculated using an unpaired *t*-test and is expressed with *p* values: grey color—no change, *p* > 0.5; green color—increase in mRNA level, *p* < 0.5; red color—decrease in mRNA level, *p* < 0.5; * *p* < 0.05; *** *p* < 0.001.

♂	2 V/m				40 V/m			
Gene	10 min	1 h	3 h	24 h	10 min	1 h	3 h	24 h
*kinin*							***	
*avpl*						*		
*at*		***				***		
*fgla*/*ast*		***	*			***		

## Data Availability

The data presented in this study are openly available in 10.6084/m9.figshare.24637908 licensed as CC BY 4.0.
